# Overview of data preprocessing for machine learning applications in human microbiome research

**DOI:** 10.3389/fmicb.2023.1250909

**Published:** 2023-10-05

**Authors:** Eliana Ibrahimi, Marta B. Lopes, Xhilda Dhamo, Andrea Simeon, Rajesh Shigdel, Karel Hron, Blaž Stres, Domenica D’Elia, Magali Berland, Laura Judith Marcos-Zambrano

**Affiliations:** ^1^Department of Biology, Faculty of Natural Sciences, University of Tirana, Tirana, Albania; ^2^Department of Mathematics, Center for Mathematics and Applications (NOVA Math), NOVA School of Science and Technology, Caparica, Portugal; ^3^UNIDEMI, Department of Mechanical and Industrial Engineering, NOVA School of Science and Technology, Caparica, Portugal; ^4^Department of Applied Mathematics, Faculty of Natural Sciences, University of Tirana, Tirana, Albania; ^5^BioSense Institute, University of Novi Sad, Novi Sad, Serbia; ^6^Department of Clinical Science, University of Bergen, Bergen, Norway; ^7^Department of Mathematical Analysis and Applications of Mathematics, Faculty of Science, Palacký University Olomouc, Olomouc, Czechia; ^8^Department of Catalysis and Chemical Reaction Engineering, National Institute of Chemistry, Ljubljana, Slovenia; ^9^Faculty of Civil and Geodetic Engineering, Institute of Sanitary Engineering, Ljubljana, Slovenia; ^10^Department of Automation, Biocybernetics and Robotics, Jožef Stefan Institute, Ljubljana, Slovenia; ^11^Department of Animal Science, Biotechnical Faculty, University of Ljubljana, Ljubljana, Slovenia; ^12^Department of Biomedical Sciences, National Research Council, Institute for Biomedical Technologies, Bari, Italy; ^13^INRAE, MetaGenoPolis, Université Paris-Saclay, Jouy-en-Josas, France; ^14^Computational Biology Group, Precision Nutrition and Cancer Research Program, IMDEA Food Institute, Madrid, Spain

**Keywords:** human microbiome, data preprocessing, machine learning, compositionality, normalization, metagenomics data

## Abstract

Although metagenomic sequencing is now the preferred technique to study microbiome-host interactions, analyzing and interpreting microbiome sequencing data presents challenges primarily attributed to the statistical specificities of the data (e.g., sparse, over-dispersed, compositional, inter-variable dependency). This mini review explores preprocessing and transformation methods applied in recent human microbiome studies to address microbiome data analysis challenges. Our results indicate a limited adoption of transformation methods targeting the statistical characteristics of microbiome sequencing data. Instead, there is a prevalent usage of relative and normalization-based transformations that do not specifically account for the specific attributes of microbiome data. The information on preprocessing and transformations applied to the data before analysis was incomplete or missing in many publications, leading to reproducibility concerns, comparability issues, and questionable results. We hope this mini review will provide researchers and newcomers to the field of human microbiome research with an up-to-date point of reference for various data transformation tools and assist them in choosing the most suitable transformation method based on their research questions, objectives, and data characteristics.

## 1. Introduction

In recent decades, next-generation sequencing technologies have significantly impacted human microbiome research, allowing for a better understanding and characterization of microbiome-host interactions (Hadrich, 2020). Numerous 16S rRNA sequencing datasets are extended further by metagenomic sequencing of the whole microbial genome. The staggering increase in publications and datasets with an ever-increasing number of samples increased the need for more performant analysis approaches, such as advanced statistical methods and machine learning (ML) algorithms that can handle large-scale microbiome datasets and extract meaningful patterns, relationships, and associations. Before entering ML analysis microbiome raw data is preprocessed through several steps shown in Supplementary Figure S1.

ML models can be trained to predict the composition of microbial communities based on various input factors such as host genetics, diet, and environmental factors, which can help us understand the factors influencing microbial composition and its relation to human health (Gupta and Gupta, 2021; Hernández Medina et al., 2022). Despite the advantages, ML analysis of microbiome data is challenging due to inherent microbiome data characteristics (i.e., sparsity, compositionality, high dimensionality, dispersion), and new techniques are requested to address these challenges (Moreno-Indias et al., 2021; D’Elia et al., 2023).

Microbiome data is zero-inflated, which can be due to the sequencing depth (i.e., sampling zeros) or the real absence of taxa (i.e., true zeros) (Silverman et al., 2020). Furthermore, variations in the abundance of one taxon affect all other taxa due to the constraint that the total counts equal the library size. Hence, the raw counts observed do not directly indicate the absolute abundances of individual taxa (Weiss et al., 2017; Lloréns-Rico et al., 2021; Swift et al., 2023), giving rise to compositional data. As a result, transforming microbiome sequencing data is essential in preparing the data for analysis and applying ML algorithms.

This mini review aims to provide a comprehensive overview of the preprocessing methods used in recent human microbiome studies to transform microbiome sequencing data before ML analysis. To collect information, we conducted a scoping review based on the methodology outlined by Arksey and O’Malley (2005), combined with manual and automated literature searches following the approach outlined by Marcos-Zambrano et al. (2021). Papers included in the final review were published in peer-reviewed journals from January 2011 to January 2022 and specifically analyzed human microbiome 16S rRNA and shotgun metagenomic data through ML algorithms. As of December 2022, 3 reviewers had extracted findings on data preprocessing and transformation techniques from 95 published studies (Supplementary Table S1). In the subsequent sections, we present and discuss the findings and outcomes of our investigation.

## 2. Sequence preprocessing

Microbiome analysis starts with raw DNA sequencing reads or microbial taxa tables at different taxonomic resolutions, from Domain (i.e., Bacteria, Archaea, Eucarya) to strain and genome variants. Microbial taxa tables are created by processing raw sequences, known as *sequence preprocessing*. Both 16S rRNA sequencing and shotgun metagenomic sequencing generally involve preprocessing steps such as quality checking, trimming, filtering, removing, and merging (Travisany et al., 2015; Ryan et al., 2020). The key differences lie in the amplification of specific gene regions for 16S rRNA sequencing and the sequencing of entire genomes for shotgun metagenomics. The sequence preprocessing steps generally depend on the origin of the DNA sequences, sequence orientation, and sequencer type.

Quality scores are used for the recognition and removal of low-quality regions of sequence (trimming) or low-quality reads (filtration) and the determination of accurate consensus sequences (merging) (Bokulich et al., 2013). A widely adopted quality metric is the Phred quality score (Q) (Galkin et al., 2020). Then, leading, and trailing trimming are applied at the position of the read where the average score drastically changes and falls below the given threshold (Bolger et al., 2014). Typical sequence preprocessing techniques are: (1) reads filtering, if overall quality is very low (Amir et al., 2017); (2) minimal length filtering, for reads below a specified length; (3) barcode and adapter-trimming (Martin, 2011); (4) chimera filtering (Edgar et al., 2011); (5) phiX reads, commonly present in marker gene of Illumina sequence data (Callahan et al., 2016). A frequently used tool for shotgun aligning and taxonomic profiling is MetaPhlAn (Thomas et al., 2019; Blanco-Míguez et al., 2023). Shotgun metagenomics preprocessing generally requires a complex sequence of programs merged into pipelines to be used since there is no one-in-all software solution yet. The solution is usually found in automated pre-defined bioBakery Workflows (Beghini et al., 2021) or Bbtools, namely, BBMerge and BBDuk (Bushnell et al., 2017; Galkin et al., 2020).

Before entering the feature selection step, additional filtering is performed on the raw data to reduce noise while keeping the most relevant taxa. In this step, microbiome low abundance features (e.g., <500 reads) and/or prevalence (e.g., <10%) per sample group or in the entire sample, are filtered out. Based on the resulting count matrix, the taxonomic level under consideration (i.e., family, genus, species) can be chosen at this stage, considering that going down to the species level would lead to strong zero inflation.

Feature selection is approached by many studies through predictive feature selection strategies that encompass statistical methods for assessing the significance of the associations between the microbiome features and the disease condition. These methods include univariate and multivariate statistical methods, and different ML algorithms (Chen et al., 2021; Jiang et al., 2022). Network-based methods have also been employed for selecting hub strains from co-occurrence networks before entering the ML task (Xu et al., 2021). It is crucial to keep in mind that when using these predictive feature selection methods, if the training dataset is not kept distinct from the test dataset throughout all preprocessing, modeling, and assessment phases, the model gains access to test set information prior to performance evaluation, resulting in data leakage (Kapoor and Narayanan, 2022). The most common ML solution for this problem is applying a cross-validation procedure, where the initial dataset is split into several folds, and in each split, different folds are proclaimed as learning or testing folds.

## 3. Transformation techniques

**Figure 1:**
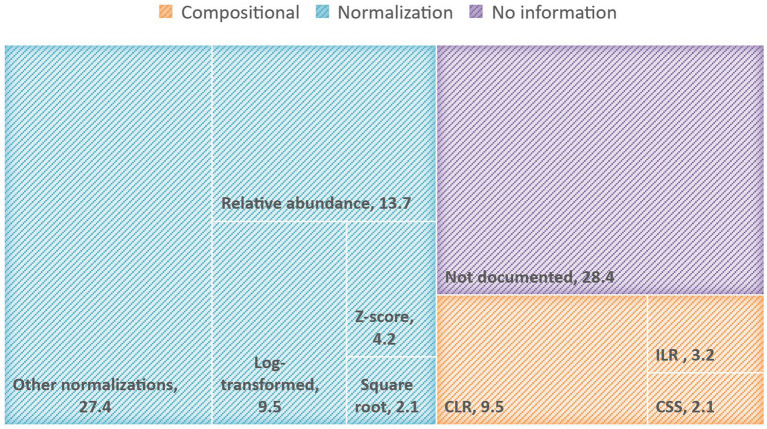
TreeMap chart illustrating the percentage of reviewed papers that applied normalization-based or compositional transformation methods, as well as the papers without clear information on preprocessing or data transformation. The other-normalization category comprises inverse-rank normalization, Box-Cox transformation, rarefaction, minimum-maximum transformation, scaling by standard deviation, normalization by total read depth, etc.

Typically, the ML analysis of microbiome data is performed after transformations are applied to raw reads to address statistical challenges mainly associated with sparsity and the proportional nature of the generated sequencing data (Lloréns-Rico et al., 2021). Based on our review, the most common data transformation methods applied in recent human microbiome studies, in both 16 s RNA sequences and shotgun data, are the relative and normalization-based methods followed by compositional transformations such as Centered log-ratio (CLR), and Isometric log-ratio (ILR). Many reviewed publications (i.e., 28%) lack sufficient details about the data preprocessing techniques that have been applied or fail to mention if any preprocessing has been carried out leading to reproducibility issues and questionable results. In Figure 1, we present a TreeMap chart illustrating the frequencies of transformation methods applied across the analyzed papers.

Within the reviewed studies, a subset dedicated to problems of disease diagnosis and risk prediction (Fabijanić and Vlahoviček, 2016; Wu et al., 2020; Ruuskanen et al., 2021; Liu et al., 2022). Data analyzed in these studies, 16S rRNA sequencing data and shotgun data, are transformed through relative abundance, log transformations, z-score normalization, and CLR. In the following subsections, we briefly discuss the normalization-based and compositional methods applied to microbiome data before ML analysis across the reviewed papers.

### 3.1. Normalization methods

Two predominant transformation methods applied to deal with uneven library sizes in sequencing microbiome data are relative abundance (Statnikov et al., 2013; Ning and Beiko, 2015; Wu et al., 2018, 2021; Bogart et al., 2019; Gupta et al., 2019; Lo and Marculescu, 2019; Vangay et al., 2019; Yachida et al., 2019; Fernández-Edreira et al., 2021; Lloréns-Rico et al., 2021), and rarefaction (Stämmler et al., 2016; Weiss et al., 2017; Baksi et al., 2018), used to solve the problem of different sequencing depths (Murovec et al., 2021).

Other normalization-based methods applied frequently to microbiome data in the reviewed studies are: Log transformation, preferred when the data is heavily skewed (Lahti et al., 2013; Fabijanić and Vlahoviček, 2016; Eck et al., 2017; Tap et al., 2017; Flemer et al., 2018; Wirbel et al., 2019; Hughes et al., 2020; Ryan et al., 2020; Fouladi et al., 2021; Jiang et al., 2021; Zhu et al., 2022). Total Sum Scaling (TSS) (Lê Cao et al., 2016; Lloréns-Rico et al., 2021) which divides each taxa count by the total number of counts in each individual sample; Minimum-Maximum normalization, used to retain the relationships between the original input data (Mulenga et al., 2021; Jiang et al., 2022); Z-score normalization (Wirbel et al., 2019; Jiang et al., 2021; Mulenga et al., 2021) which transforms the data with mean zero and unit variance; the Square Root that can be successfully applied to count data that follow a Poisson distribution (Liu et al., 2011; Holmes et al., 2012); Inverse-Rank normalization used to normalize signals to approximate a normal distribution after removing the quality control sample (Ni et al., 2021).

### 3.2. Compositional transformations

Our review reveals a noticeable rise in the utilization of ML techniques within human microbiome research over recent years, while the adoption of compositional transformations in handling microbiome data remains relatively constrained. Nevertheless, an encouraging increasing trend in the application of compositional approaches between 2016 and 2021 is observed, as visually represented in Supplementary Figure S2. The following paragraphs delve into compositional transformations that have been employed in recent human microbiome studies, while in Table 1 we provide an overview of the relevant literature and software tools necessary for the successful implementation of these methods.

**Table 1:** Compositional transformations that are applied to human microbiome 16S rRNA and shotgun data.

Method	Bioconductor/R package	Literature
Additive log-ratio	Compositions	Aitchison (1982, 1986) and van den Boogaart and Tolosana-Delgado (2008)
Centered log-ratio	Compositions	Pawlowsky-Glahn et al. (2015) and van den Boogaart and Tolosana-Delgado (2008)
Isometric log-ratio	Compositions	Egozcue et al. (2003) and van den Boogaart and Tolosana-Delgado (2008)
Geometric mean of pairwise ratios	GMPR	Chen et al. (2018)
Trimmed mean of M-values	edgeR	Robinson et al. (2010)
Relative log expression (RLE)	edgeR	Robinson et al. (2010)
Variance-stabilizing (VST)	DESeq2	Love et al. (2014)

Compositional data can be represented in a simplex space and analyzing them as absolute data with standard statistical techniques may lead to inappropriate results (Gloor et al., 2016; Quinn et al., 2018). Aitchison (1982) first proposed the additive log-ratio transformation (ALR), to address compositionality then also the centered log-ratio (CLR) (Aitchison, 1986). His followers proposed further the isometric log-ratio (ILR) (Egozcue et al., 2003; Pawlowsky-Glahn et al., 2015) and pivot log-ratio (PLR) (Filzmoser et al., 2018) transformations. The CLR transformation is applied more frequently in microbiome studies (Fabijanić and Vlahoviček, 2016; Lê Cao et al., 2016; Wirbel et al., 2019; Fukui et al., 2020; Reiman et al., 2021; Ruuskanen et al., 2021; Liu et al., 2022) than the ILR transformation (Kubinski et al., 2022), while the ALR was not applied in any of the studies included in the review.

Other compositional transformations that can be applied in microbiome data are: Cumulative Sum Scaling (CSS) (Dhungel et al., 2021; Lloréns-Rico et al., 2021), a particular representation of the relative information based on median-like quantiles; the Geometric mean of pairwise ratios (GMPR) transformation (Chen et al., 2018); the Trimmed mean of M-values (TMM) (Robinson et al., 2010); the Relative log expression (RLE) method (Robinson et al., 2010); the Variance-stabilizing transformation (VST) (Love et al., 2014).

## 4. Discussion

Transformations are essential for appropriately handling microbiome sequencing data, rectifying compositional issues, reducing noise, adhering to statistical assumptions, and enabling meaningful analysis and interpretation. The choice of transformation should depend on the specific characteristics of the data and the goals of the analysis. This mini review revealed substantial gaps in the process of microbiome data transformation. Relative transformations and other normalization-based methods that lead to or do not solve compositional issues (Lloréns-Rico et al., 2021) are frequently applied in recent human microbiome research.

Unlike compositional approaches (i.e., log ratios), normalization-based methods do not retrieve absolute scale from the relative data (Quinn et al., 2018). Nevertheless, when the raw data contains zero values, like in microbiome data, taking the logarithm results in negative infinity, distorting the data, and leading to invalid statistical inferences. To mitigate this issue, a pseudocount (i.e., small positive constant, ε) can be added to zero values before taking the logarithm. Selecting the right pseudocount in relation to the data’s scale holds significant importance when applying log transformations (Thorsen et al., 2016). The scale of the ε, relative to the total read counts, should remain consistent across different data transformation methods applied (McKnight et al., 2019) and should be based on the context of the research problem and the scale of the data because the choice of ε can affect the results (Costea et al., 2014). Thus, it is essential to be mindful of the trade-offs between numerical stability and introducing additional bias due to the choice of ε.

Compositional transformations, ALR, CLR, and ILR log-ratio transformations, have different properties. The ALR transformation does not preserve distances because it is not isometric (Egozcue and Pawlowsky-Glahn, 2005), while CLR transformation keeps the distance, but the covariance and correlation matrix are singular because of the zero-sum of the transformed vectors (Quinn et al., 2018). In addition, aggregation of all components into the geometric mean can, in general, lead to the occurrence of false positives (Filzmoser and Walczak, 2014), so identifying the original components with the corresponding CLR variables has some limitations, which could possibly be overcome by a proper weighting strategy (Štefelová et al., 2021). Recent studies suggest that for high-dimensional compositional data, the ALR transformation should be a preferred choice for transforming variables because the interpretation of ALRs is easier than the ILR and CLR transformations (Greenacre et al., 2021). Besides log ratios, other transformations such as VST and ranked-based methods have been reported to successfully address microbiome data statistical specificities (Jeganathan and Holmes, 2021; Lloréns-Rico et al., 2021). When working with spatial human microbiome data, which can reflect the microbial composition and abundance within specific locations in the body (Adade et al., 2021), transformations for compositional spatial data that would improve ML techniques’ performance when dealing with this data can be considered. Greenacre (2010, 2011) explored a power transformation that converges toward the Aitchison log-ratio transformation when the power parameter becomes 0, while Clarotto et al. (2022) propose the Isometric α-transformation (α-IT), which, unlike the ILR transformation, can successfully deal with zeros in the data.

Kubinski et al. (2022) investigated the impact of various transformation techniques on the model’s predictive performance using gut microbiome data and highlighted the need to transform 16S rRNA data using compositional transformation techniques. Among the available options, the CLR transformation was identified as the most suitable, as it enables the assessment of each feature’s importance in the decision-making process of ML models. Another study by McKnight et al. (2019) examined the impact of log transformations commonly employed in normalization procedures. The authors demonstrated that log transformations could distort community comparisons by suppressing significant differences in common taxa while amplifying subtle differences in rare taxa.

Thus, despite the advantages, log-ratio approaches have their limitations and drawbacks and are not the only way to deal with compositionality. Quantitative transformations such as Quantitative Microbiota Profiling (QMP) (Vandeputte et al., 2017) and Absolute Counts Scaling (ACS) (Props et al., 2017; Jian et al., 2020) offer experimental approaches to address microbiome data proportional nature. QMP involves rarefying samples to achieve an even sampling depth and scaling them based on estimated microbial loads. On the other hand, ACS directly scales the relative sequencing counts using estimated microbial loads. Lloréns-Rico et al. (2021) investigated the impact of computational and experimental techniques in addressing the issues arising from microbiome data features (i.e., compositionality and sparsity). They concluded that quantitative approaches outperform computational methods in addressing compositionality and sparsity. Authors claim that the quantitative approaches improve the identification of true positive associations while reducing the occurrence of false positives. The same study reports that when adopting quantitative methods is not feasible, computational methods that address compositionality perform better than relative methods. There are other examples in the literature where compositional methods are employed to transform microbiome data where the reader can find more details (Quinn and Erb, 2020; Yang and Zou, 2020; Greenacre et al., 2021; Yang et al., 2021; Papoutsoglou et al., 2023).

It is important to mention that in many cases the analysis of microbiome data can be performed on raw read counts rather than in transformed data. Zero-inflated negative binomial and Dirichlet-multinomial models can fit microbiome raw data quite well (Xia et al., 2018). For example, Zhang et al. (2017) applied on raw read counts a negative binomial mixed model that enables the identification of connections between the host, environmental variables, and the microbiome.

Finally, the lack of adequate information on data preprocessing and high reporting heterogeneity among papers highlight the need for standardized reporting guidelines, as also suggested by Mirzayi et al. (2021), where recommendations and guidelines are provided to help microbiome researchers properly report their findings through the ‘Strengthening The Organization and Reporting of Microbiome Studies’ (STORMS), composed of a 17-item checklist each related with the typical sections of a scientific paper. The omission of preprocessing and transformations applied to the data can have several significant consequences such as reproducibility concerns, misinterpretation, comparability issues, and questionable results. To mitigate these consequences, it is essential for researchers to provide thorough documentation of their data preprocessing procedures in publications. Researchers should also consider sharing their code, scripts, or workflows used for data preprocessing, which can greatly enhance transparency and reproducibility.

## 5. Conclusions and final remarks

Our short review shows that the utilization of data transformations that address the proportional nature of microbiome sequencing data in human microbiome studies remains limited, with many researchers primarily opting for relative and normalization-based methods that do not specifically address microbiome data characteristics. There is a lack of transparency and clear explanations regarding data preprocessing and the choice of transformation methods among the reviewed papers while it is crucial to adhere to best practices and provide a detailed methodology for developing machine learning pipelines, particularly regarding data preprocessing.

This mini review does not intend to provide unequivocal recommendations in favor of one approach over another, instead, we encourage researchers to consider the characteristics of their data carefully and whether a particular transformation method is suitable for addressing their research questions and data characteristics.

## Author contributions

EI: conceptualization, investigation, writing the draft and the final manuscript. ML: investigation and writing the draft and final manuscript. XD and AS: writing the draft manuscript. RS: investigation. KH, BS, DD’E, and MB revised the draft manuscript, provided comments and writing the final manuscript. LM-Z: conceptualization, investigation, and writing the draft and final manuscript. All authors contributed to the article and approved the submitted version.
