# Supplementary material for: Overview of data preprocessing for machine learning applications in human microbiome research
Source: Front Microbiol. 2023 Oct 5;14:1250909. doi: 10.3389/fmicb.2023.1250909 (PMC10588656; doi:10.3389/fmicb.2023.1250909)
Supplement: Supplementary file 2 [file Data_Sheet_1.pdf]

Supplementary Material

Overview of Data Preprocessing for Machine Learning Applications in Human Microbiome Research

Eliana Ibrahimi[1]*, Marta B. Lopes[2,3], Xhilda Dhamo[4], Andrea Simeon[5], Rajesh Shigdel[6], Karel Hron[7], Blaž Stres[8,9,10,11], Domenica D'Elia[12], Magali Berland[13], Laura Judith Marcos-Zambrano[14]*

*** Correspondence:** Corresponding Author: eliana.ibrahimi@fshn.edu.al; judith.marcos@imdea.org

1 Supplementary Figures and Tables

1.1 Supplementary Figures

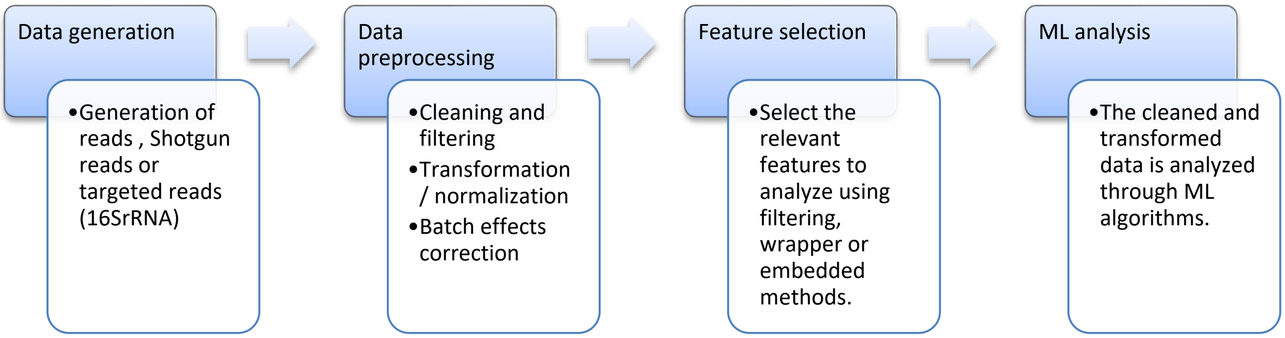

Supplementary Figure 1. Flowchart illustrating the preprocessing steps for microbiome data.

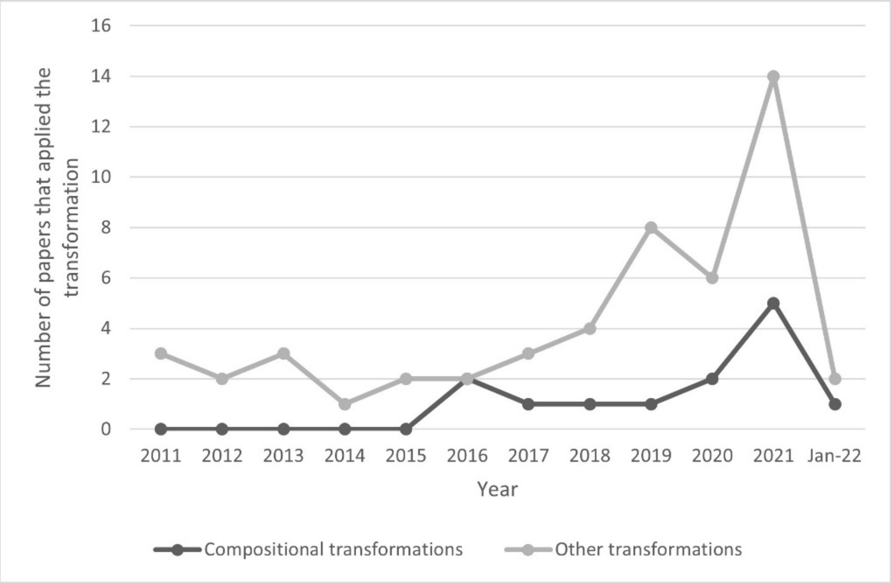

Supplementary Figure 2. Compositional transformations applied to human microbiome data versus other transformation techniques by the time of publication.

1.2 Supplementary Tables

Supplementary Table 1: List of reviewed papers and transformation method applied.
